# Notes and News

**Published:** 1890-11-01

**Authors:** 


### Notes and News.

Collected and Communicated.

A very successful concert has been held in an empty ward of Torbay Hospital, Torquay, for the benefit of the funds of the hospital, by which £18 8s. was handed to the chairman. Expenses were only £2 15s. Everyone gave their services, amongst whom were three professional gentlemen and several well-known amateurs. Plants were lent by Madame Capinette. Everyone was kind and gracious, and all passed off with great *éclat*. It is hoped to give a concert for the patients before long.

The committee of the Caxton Seaside Convalescent Home and Sanatorium have elected Swanage, in Dorset, as the most suitable place wherein to erect a building for their requirements. This course has been resolved upon after an exhaustive and searching inspection had been made of properties at Worthing, Milford, Boscombe, and other places. At one time it was hoped that a particular property at Swanage would have been found suitable, but expert opinion, that of Mr. A. Saxon Snell, the architect, reported unfavourably of its adaptability for the proposed object. The committee exercised a judicious discrimination in deciding it would be more economical in the end to erect a building in accordance with modern scientific knowledge than to negotiate for an existing edifice which would need extensive alteration, possibly never perfect, and not meet with the approval of the faculty. Dr. Forbes Winslow, physician to the North London Consumptive Hospital, says of Swanage that "statistics have proved that Swanage is the coolest summer residence on the south coast, and is another proof of its fitness for invalids afflicted with other complaints besides those influencing the respiratory or circulatory systems. It possessed the warmest night temperature in England (minimum), except Ilfracombe, Ventnor, and Maker." Dr. B. Ward Richardson and Dr. William Miller Orde also speak favourably of Swanage as a place for convalescents. It is to be hoped that this committee will receive the practical support of employers and those interested in the printing and kindred trades, as they have of their fellow-craftsmen, in bringing their philanthropic labours to a successful issue at an early date. The treasurer is Mr. C. W. Bowerman (secretary News Branch, L.S.C.), 19, Ronald's Road, Highbury, N.; and the bankers, the Birkbeck Bank, Southampton Buildings, Chancery Lane, W.C.

Scrap-books and presents for the Leper Hospital a Colombo, to which we referred in our issue of October 11th, can be sent to Miss Gethen, 55, Burton Crescent, W.C., who will forward them to the chaplain.

The Duke of Portland this week opened a new hospital at Mansfield, which has been erected in commemoration of Her Majesty's jubilee. His Grace gave a cheque for £300 to the endowment fund, and asked to be allowed to contribute the cost of one bed in the hospital on behalf of his wife. The Duchess, he stated, was much better than before she went to Scotland, and she hoped in a short time to take part in public duties.

A Melbourne doctor describes the general hospital as only fit to be burnt down, because the bricks are so porous you can blow out a candle held on the other side of one of the dividing walls. The place, he says, is saturated with germs. Then comes another doctor, who describes a hospital with tiled walls and polished floors; there is no proper ventilation there, cries the new prophet; as well varnish a man all over and expect him to live, as expect a sick man to recover in an impermeable ward. Such is life! always two sides to a question, and always two parties ready for strife!

Sequah is at present attracting large crowds twice daily at Richmond. The proceedings are rather slow in spite of a brass band, for there is a long lecture before and after the rubbing of the patient, and the lecture is all in praise of the oil and the medicine. The most marvellous tales of cures are of course told by the poor of the neighbourhood, and the shillings drop gaily into the hat of the man who sells the wondrous remedies. Sequah—or the man who goes by that name, for it seems that there are at least a dozen similar men travelling the country—is a good actor and orator, and ostentatiously charitable. We can quite understand that with such agents the Sequah Company can pay a big dividend.

The Victoria Hospital for Sick Children at Hull issues its seventeenth annual report, and the committee congratulate themselves on the progress of the new hospital. The total number of in-patients treated was 274, the out-patients 2,280. Over 80 patients enjoyed the benefit of the Convalescent Home at Hornsea, and the Samaritan Fund has helped those children needing instruments. A page of the report is devoted to the memory of the ex-chairman, Mr. Henry Allison, who died suddenly last June. The present Mayor of Hull, Dr. Sherburn, is consulting surgeon, and was once house surgeon to the Victoria Hospital, and ever maintains the greatest interest in the institution. At his suggestion, the town has given the hospital a Jubilee offering of a life-like portrait of the Queen.

The *Guy's Hospital Gazette* publishes the following excellent points made by Mr. G. A. Wright in the course of his paper read before the Guy's Hospital Medical Society the other day. Speaking of the hospitals in the North, Mr. Wright said: "It is, no doubt, often difficult for laymen to appreciate the aims and needs of the medical workers, and it requires an educated and thoughtful man to see that a hospital is not merely a place where the annual report must be made to show the largest possible number of patients treated for the smallest possible sum per head. In Manchester we are happily gradually emerging from the dark days when medical students were looked upon as hardly necessary evils, and their existence was supposed to be a concession to the interest of the medical staff. I am glad to say that people now pretty generally recognise the fact that the best guarantee for the welfare of hospital patients is the presence of a medical school in connection with the hospital."

THE following vacancies are announced this week, the amount of the salary in each case being given after the name of the institution:—

**Resident Medical Officers.**

Victoria Park Chest Hospital, House Physician—board, &c.
Radcliffe Infirmary, Oxford, House Surgeon—£80, board, &c.
Manchester Clinical Hospital for Women, House Surgeon, £80—board, &c.
Cape of Good Hope Government, Bacteriologist—£500.
Cape of Good Hope Government, Toxicologist—£400.

**Non-Resident Medical Officers.**

Scarborough Friendly Societies, Assistant Medical Officer—£140.
Victoria Park Chest Hospital, Pathologist—£100.
University College, Jodrell Professor of Comparative Anatomy and Zoology.
King's College Hospital, Assistant Physician.
King's College Museum, Senior Attendant.
Victoria Hospital for Sick Children, Assistant Physician.
Victoria Hospital for Sick Children, Assistant Surgeon.

---

AN amusing tale appears in a local paper about the West London Hospital. Some time since the daily papers told of an accident to a small boy who was run over in trying to save a cat. A tender-hearted lady sent a handsome sum for the humane little fellow, who had been injured in the cause of a poor dumb animal. Imagine the feelings of the lady when the matron courteously acknowledged the money, but stated that it was a *tip-cat* the boy had tried to save!

---

THE *Lancet* tells us ladies should not wear combs in their hair, or pins in their hats—they might fall down and hurt themselves with the combs or the pins. The *Daily News* tells us we are not to keep lap-dogs, less they lick us and give us cysticercus of the liver. Is life worth living? No, certainly not, if we are always dreading disease and death; and there is an old saying, "Evils come to those who fear them." But perhaps the sharpest sarcasm ever hurled at these too cautious creatures was that of a living Scotch novelist, who remarked, "Dear me, Professor, I hope you never go to bed; I am not well acquainted with these dangers of which you speak, but I am absolutely convinced that more people die in their beds than elsewhere. I must beg, Professor, that you never indulge in such a dangerous procedure as going to bed."

---

OUR contemporary, the *Lancaster Guardian*, has been hammering away, and with reason, at the Infirmary Committee in Lancaster. According to our contemporary there is £13,000 available for a new infirmary of 50 beds, but no steps are to be taken to erect the new buildings unless, or until, £21,000 is contributed, that being the lowest sum, so the committee contend, at which an infirmary can be built for 50 patients. There must surely be some mistake somewhere, as we cannot credit that any committee would be so foolish as to contend that it is either necessary or desirable to spend anything like £420 per bed upon buildings when a new hospital has to be erected. A very compact and useful hospital for 50 beds, replete with the latest and most perfect sanitary and hygienic arrangements, including the furniture, could be erected in Lancaster for from ten to twelve thousand pounds. In these circumstances we should be glad to have an explanation of the points at issue. The present buildings only contain 33 beds, of which only 22 on an average are constantly occupied. Lancaster does not, therefore, need a larger infirmary than one of 50 beds at present, at any rate, and those who contend to the contrary, if any such there be, should visit Gloucester or Stratford-on-Avon, or the Johnson Hospital, Spalding, and learn the cost of maintaining extra buildings not required for the continuous occupation of patients.

